# PORCN Negatively Regulates AMPAR Function Independently of Subunit Composition and the Amino-Terminal and Carboxy-Terminal Domains of AMPARs

**DOI:** 10.3389/fcell.2020.00829

**Published:** 2020-08-25

**Authors:** Mengping Wei, Meng Wang, Jue Wang, Feng Su, Yangzhen Wang, Meng Sun, Shanshan Wang, Mengna Liu, Hongyi Wang, Mingyang Lu, Wei Li, Yutian Gong, Lei Yang, Chen Zhang

**Affiliations:** ^1^PKU-IDG/McGovern Institute for Brain Research, School of Life Sciences, Peking University, Beijing, China; ^2^Beijing Key Laboratory of Neural Regeneration and Repair, School of Basic Medical Sciences, Advanced Innovation Center for Human Brain Protection, Capital Medical University, Beijing, China; ^3^Peking-Tsinghua Center for Life Sciences, Academy for Advanced Interdisciplinary Studies, Peking University, Beijing, China; ^4^School of Life Sciences, Tsinghua University, Beijing, China

**Keywords:** AMPA receptor, PORCN, receptor trafficking, glutamate-induced currents, protein-protein interactions

## Abstract

Most fast excitatory synaptic transmissions in the mammalian brain are mediated by α-amino-3-hydroxy-5-methylisoxazole-4-propionic acid receptors (AMPARs), which are ligand-gated cation channels. The membrane expression level of AMPARs is largely determined by auxiliary subunits in AMPAR macromolecules, including porcupine O-acyltransferase (PORCN), which negatively regulates AMPAR trafficking to the plasma membrane. However, whether PORCN-mediated regulation depends on AMPAR subunit composition or particular regions of a subunit has not been determined. We systematically examined the effects of PORCN on the ligand-gated current and surface expression level of GluA1, GluA2, and GluA3 AMPAR subunits, alone and in combination, as well as the PORCN-GluA interaction in heterologous HEK293T cells. PORCN inhibited glutamate-induced currents and the surface expression of investigated GluA AMPAR subunits in a subunit-independent manner. These inhibitory effects required neither the amino-terminal domain (ATD) nor the carboxy-terminal domain (CTD) of GluA subunits. In addition, PORCN interacted with AMPARs independently of their ATD or CTD. Thus, the functional inhibition of AMPARs by PORCN in transfected heterologous cells was independent of the ATD, CTD, and subunit composition of AMPARs.

## Introduction

Most fast excitatory synaptic transmissions in the mammalian brain are mediated by α -amino-3-hydroxy-5-methylisoxazole-4-propionic acid receptors (AMPARs), which are ligand-gated cation channels. Dynamic changes in AMPAR properties serve as a major mechanism governing many forms of synaptic plasticity, including homeostatic scaling and long-term depression and potentiation (Malenka, 2003; Kessels and Malinow, 2009; Huganir and Nicoll, 2013; Diering and Huganir, 2018). AMPAR dysfunction results in neuropsychiatric diseases, such as Alzheimer’s disease (Madsen et al., 1994; Whitehead et al., 2017), schizophrenia (Danysz, 2002; Barkus et al., 2014; Ward et al., 2015), and autism (Sudhof, 2008; Lee et al., 2016; Cheng et al., 2017). Mature AMPARs are tetramers comprising different combinations of four pore-forming subunits, which are as follows: GluA1, GluA2, GluA3, and GluA4 (Wu et al., 1996; Rosenmund et al., 1998; Dingledine et al., 1999; Greger and Mayer, 2019). Each GluA subunit comprises a large extracellular amino-terminal domain (ATD), three transmembrane domains (M1, M3, and M4), one reentrant loop (M2), a ligand-binding domain (LBD), and a carboxy-terminal domain (CTD). In addition, the endogenous AMPAR complex contains multiple auxiliary subunits that, together with the GluA tetramer, form a 0.6 to 1 MDa macromolecule (Schwenk et al., 2012). Genetic and proteomic studies have revealed more than a dozen auxiliary subunits, including transmembrane AMPAR regulatory proteins [TARPs; (Chen et al., 1999, 2000; Hashimoto et al., 1999; Tomita et al., 2003; Rouach et al., 2005)], cornichon homolog 2/3 [CNIH-2/3; (Schwenk et al., 2009; Kato et al., 2010; Herring et al., 2013)], germ cell-specific gene 1-like protein [GSG1L; (Shanks et al., 2012; McGee et al., 2015; Gu et al., 2016)], abhydrolase domain containing 6 [ABHD6; (Wei et al., 2016, 2017)], and porcupine O-acyltransferase [PORCN; (Erlenhardt et al., 2016)]. These auxiliary subunits modulate membrane localization, synaptic targeting, interorganelle trafficking, and the channel kinetics of AMPARs [reviewed in Jackson and Nicoll (2011), Cheng et al. (2012), Straub and Tomita (2012), Bettler and Fakler (2017), Bissen et al. (2019)].

As transmembrane receptors, AMPARs are transported to the plasma membrane, where they bind glutamate transmitters to transmit signals from the presynaptic neuron to the postsynaptic neuron. The auxiliary subunits of AMPARs are important mediators of AMPAR cell surface localization. Auxiliary subunits, including TARPS, CNIH-2/3, GSG1L, ABHD6, and PORCN, have been shown to have a role in the AMPAR trafficking process. Some auxiliary subunits, like stargazin and other type-I TARPS (Chen et al., 2000; Rouach et al., 2005; Straub and Tomita, 2012; Ben-Yaacov et al., 2017), promote AMPAR trafficking to the membrane and consequent synaptic transmission, whereas other auxiliary subunits, like GSG1L (Shanks et al., 2012; McGee et al., 2015; Gu et al., 2016) and ABHD6 (Wei et al., 2016, 2017), reduce the surface level of AMPARs and, hence, AMPAR-mediated excitatory neurotransmission. The auxiliary subunit PORCN serves as a negative regulator for AMPAR function in both neuronal cells and transfected heterologous cells (Erlenhardt et al., 2016). In this study, the inactivation of PORCN in hippocampal neurons reduced the amplitude but accelerated the decay kinetics of AMPAR-mediated synaptic transmission. Additionally, biochemistry analysis revealed a significant reduction in GluA1 and GluA2/3 levels in crude extracts and intracellular membrane fractions. However, there was a significant reduction in GluA2/3 but not GluA1 expression levels in the postsynaptic density (PSD) fraction. Finally, in HEK293T cells that lacked expression of AMPARs, PORCN overexpression decreased glutamate-induced currents when exogenously expressing GluA1 homologous AMPARs (Erlenhardt et al., 2016). Whether the inhibitory effects of PORCN on AMPAR function involve interactions with a specific AMPAR subunit or particular regions of an AMPAR subunit has not been determined.

In the present study, we examined the AMPAR subunit requirement for PORCN-mediated inhibition of AMPAR function in transfected heterologous cells. We showed that PORCN inhibits glutamate-induced currents and AMPAR surface expression in an AMPAR subunit-independent manner in heterologous cells. Furthermore, the ATD and CTD of AMPARs were not required to mediate the inhibitory effect of PORCN. We used immunoprecipitation assays to show that PORCN associated with all AMPAR subunits independently of the ATD and CTD. This was consistent with the functional data. Thus, our observations strongly supported the hypothesis that PORCN regulates AMPAR trafficking to the plasma membrane through protein-protein interactions.

## Materials and Methods

### HEK293T Cell Culture and Transfection

In this study, stargazin, PORCN, full-length GluA subunits, and GluA deletion constructs were expressed in HEK293T cells (CRL-11268, ATCC). First, cells were cultured in a 37°C incubator supplied with 5% CO_2_. Then, cells were dissociated with 0.05% trypsin and plated on dishes at a density of 800,000 cells per 35 mm dish (counted with a μScope CellCounter Basic; C.E.T.) 24 h before transfection. A 2:3 ratio of GluA:stargazin cDNA and a 3:1 ratio of GluA-stargazin:PORCN cDNA was used as previously reported (Shi et al., 2009; Wei et al., 2017). A cDNA ratio of 3:2 was used for the coexpression of GluA1 and GluA2 as well as GluA2 and GluA3 as previously reported (Shi et al., 2009; Wei et al., 2017). In control groups, the same amount of empty vector was used instead of PORCN cDNA. Transfection was performed using polyethylenimine (Polysciences, United States) reagents. Transfected HEK293T cells were dissociated with 0.05% trypsin and plated on pretreated coverslips that were 8 mm diameter and coated with poly-D-lysine. Electrophysiological recording or immunostaining analyses were performed on cells transfected with 4 μg of total cDNA per 35 mm dish 24–36 h after transfection. For Western blotting, cells were transfected with 6.75 μg of full-length GluA subunit or GluA deletion plasmids together with 2.25 μg of myc-PORCN or control plasmids in 60 mm dishes and harvested 48 h after transfection.

### Electrophysiological Recording

Electrophysiological recording was conducted on coverslips seeded with transfected HEK293T cells maintained in an external solution of 144 mM NaCl, 10 mM KCl, 2 mM CaCl_2_, 1 mM MgCl_2_, 10 mM HEPES, and 10 mM D-glucose (pH 7.3–7.4, Osm 315 mOsm/kg). For whole-cell patches, microelectrodes (3–5 MΩ, World Precision Instruments) were filled with an internal solution of 145 mM KCl, 5 mM NaCl, 5 mM EGTA, 4 mM MgATP, 0.3 mM Na_2_GTP, and 10 mM HEPES (pH 7.2, Osm 305 mOsm/kg). Series resistance was compensated to 60–70%, and recordings with series resistance values greater than 20 MΩ were rejected. Glutamate-induced currents were elicited through the local administration of external solution containing 10 mM L-glutamate acid (Sigma, G8415) for 2 s using an MPS-2 perfusion instrument [Inbio Life Science Instrument Co., Ltd.; (Wu et al., 2005)]. Whole-cell voltage clamp recordings were taken with an EPC10 patch clamp amplifier (HEKA, Lambrecht, Germany). Data were analyzed using the following software packages: Clampfit 10.0 (pClamp), Prism 5 (GraphPad Prism), and Igor 6.02 (WaveMetrics).

### Hippocampal Culture and Calcium-Phosphate Transfection

Hippocampi were dissected from P0 pups and digested with 0.25% trypsin (Gibco, 25200072) at 37°C for 15 min. Neurons were plated on poly-D-lysine-coated glass coverslips and maintained at 37°C in 5% CO_2_ for 14 days before the experiment. The calcium-phosphate transfection method was used for the transfection of cultured neurons at 10 days *in vitro*. The DNA (0.5 μg per well in a 48 well plate) and Ca^2+^ were mixed and added to HBS drop by drop with a gentle vortex. After keeping the DNA/Ca^2+^/HBS mixture at room temperature for 30 min, it was added to the culture and incubated for 40 min at 37°C. Then, the culture was washed with culture medium two to three times and kept in the incubator.

### Immunostaining

Immunostaining analyses were performed as previously described (Jiang et al., 2017). In brief, transfected HEK293T cells were washed once with phosphate-buffered saline (PBS; Thermo Scientific), fixed with 4% paraformaldehyde in PBS for 10 min at room temperature, and washed three times with PBS. Then, cells were permeabilized with 0.2% Triton X-100 for 5 min at room temperature for total protein analysis or were left unpermeabilized for surface protein analysis. After blocking with PBS containing 5% milk and 3% goat serum for 30 min at room temperature, cells were incubated with a primary antibody (anti-HA, 1:1000, Abmart; anti-Flag, 1:1000, Abmart) for 2 h at room temperature, washed three times with PBS, and incubated with the secondary antibody (donkey anti-mouse Alexa Fluor 546-conjugated secondary antibody, Life Technologies) for 30 min at room temperature. Fluoromount-G (Southern Biotech) was used to mount the cells on microscope slides. Images were acquired with a laser scanning confocal microscope (Olympus, FV3000) using a 60× objective lens (Olympus) and were further analyzed using the National Institutes of Health ImageJ program and Prism 5 software (GraphPad Prism).

### Immunoblotting and co-IP Assay

Transfected HEK293T cells were washed once with PBS and incubated in 360 μL of cell lysis buffer comprising 50 mM Tris–HCl, 1 mM EDTA, 150 mM NaCl, and 1% CA-630 for 15 min at 4°C. Then, cell lysates were collected and centrifuged at 12,000 × *g* for 10 min at 4°C to remove the insoluble fraction. The supernatant was collected, and 10 μL was used as the input, while 350 μL was used for co-IP. Anti-myc magnetic beads (40 μL, 88843, Thermo Scientific) were added to the samples and rotated for 12 h at 4°C then washed four times with cell lysis buffer comprising 50 mM Tris–HCl, 1 mM EDTA, 150 mM NaCl, 1% lgepal CA-630, pH 7.4. Input and pulldown beads were heated at 70°C in sample buffer comprising 4× lithium dodecyl sulfate sample buffer and 10× sample reducing buffer. Then, they were subjected to SDS-PAGE (10% Bis-Tris gels, Life Technology) for 45 min at 200 V and transferred to nitrocellulose membranes. After blocking in SuperBlock T20 blocking buffer (37516, Thermo Scientific), membranes were incubated overnight at 4°C with primary antibodies against GluA1 (AB1504, Millipore, 1:2000), GluA2 (13607, CST, 1:2000), and flag (AE004, Abclonal, 1:1000). After three washes, membranes were incubated with the secondary antibody (IRDye 680LT goat anti-mouse IgG and 800CW goat anti-rabbit IgG, Odyssey) for 1 h at room temperature. Signals were detected with an infrared imaging system (Odyssey) and analyzed using the National Institutes of Health ImageJ program and Prism 5 software (GraphPad Prism).

## Results

### PORCN Suppressed Glutamate-Induced Currents in HEK293T Cells Expressing GluA1, A2, or A3 With or Without Stargazin

**FIGURE 1:**
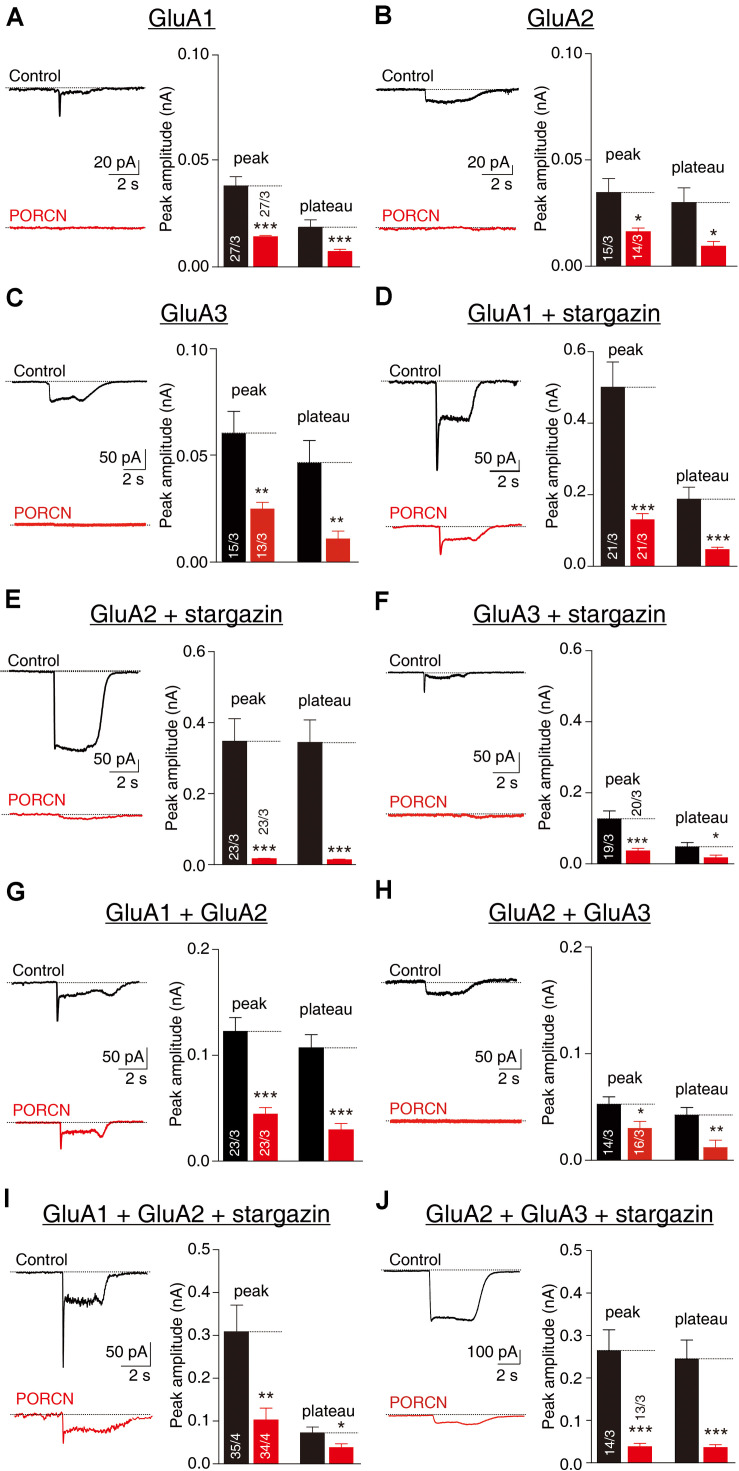
The overexpression of PORCN suppressedglutamate-induced currents mediated by GluA2 and GluA3 in HEK293T cells with or without stargazin coexpression. **(A)** Representative traces (left) and summary graphs (right) of the peak amplitudes and plateaus of 10 mM glutamate-induced currents in HEK293T cells transfected with GluA1 and either PORCN or a control plasmid. **(B)** Representative traces (left) and summary graphs (right) of the peak amplitudes and plateaus of 10 mM glutamate-induced currents in HEK293T cells transfected with GluA2 and either PORCN or a control plasmid. **(C)** Representative traces (left) and summary graphs (right) of the peak amplitudes and plateaus of 10 mM glutamate-induced currents in HEK293T cells transfected with GluA3 and either PORCN or a control plasmid. **(D)** Representative traces and summary graphs of the peak amplitudes and plateaus of 10 mM glutamate-induced currents in HEK293T cells transfected with GluA1, stargazin, and either PORCN or a control plasmid. **(E)** Representative traces and summary graphs of the peak amplitudes and plateaus of 10 mM glutamate-induced currents in HEK293T cells transfected with GluA2, stargazin, and either PORCN or a control plasmid. **(F)** Representative traces and summary graphs of the peak amplitudes and plateaus of 10 mM glutamate-induced currents in HEK293T cells transfected with GluA3, stargazin, and either PORCN or a control plasmid. **(G)** Representative traces and summary graphs of the peak amplitudes and plateaus of 10 mM glutamate-induced currents in HEK293T cells transfected with GluA1 and GluA2 and either PORCN or a control plasmid. **(H)** Representative traces and summary graphs of the peak amplitudes and plateaus of 10 mM glutamate-induced currents in HEK293T cells transfected with GluA2 and GluA3 and either PORCN or a control plasmid. **(I)** Representative traces and summary graphs of the peak amplitudes and plateaus of 10 mM glutamate-induced currents in HEK293T cells transfected with GluA1, GluA2, and stargazin and either PORCN or a control plasmid. **(J)** Representative traces and summary graphs of the peak amplitudes and plateaus of 10 mM glutamate-induced currents in HEK293T cells transfected with GluA2, GluA3, and stargazin and either PORCN or a control plasmid. In all panels, the black traces and bars represent the control condition (no PORCN expression), while the red traces and bars represent PORCN overexpression. All summary graphs show means ± SEMs; statistical comparisons were performed with a student’s *t*-test (*p < 0.05; **p < 0.01; ***p < 0.001).

To investigate whether the functional inhibition of AMPARs by PORCN depended on AMPAR subunit composition, we measured glutamate-induced currents via the whole-cell patch clamping of HEK293T cells transfected with various AMPAR subunits alone or in combination with stargazin and/or PORCN. Stargazin was used to promote AMPAR cell surface localization (Chen et al., 2000). Glutamate-induced currents were undetectable in HEK293T cells without transfection, because such cells do not normally express AMPAR subunits (Wei et al., 2016). In this study, PORCN expression significantly suppressed glutamate-induced currents mediated by GluA1 (Figure 1A), GluA2 (Figure 1B), and GluA3 (Figure 1C). The coexpression of stargazin with GluA2 or GluA3 significantly increased glutamate-induced currents compared with GluA2 or GluA3 expression alone but did not abolish the PORCN-mediated inhibition of AMPAR-mediated currents (Figures 1D–F). PORCN expression inhibited the peak amplitude of glutamate-induced currents in GluA1, GluA2, and GluA3 overexpressing cells with stargazin coexpression by 74.14 ± 14.79%, 95.39 ± 18.52%, and 70.99 ± 18.34%, respectively, and without stargazin coexpression by 60.45 ± 15.65%, 53.85 ± 20.45%, and 58.55 ± 19.44%, respectively. PORCN had similar effects on the plateau amplitude of glutamate-induced currents. In this case, percentage inhibition with stargazin coexpression was 75.39 ± 18.88%, 96.13 ± 18.72%, and 63.90 ± 30.36%, and without stargazin coexpression, it was 56.00 ± 15.30%, 68.14 ± 25.46%, and 76.37 ± 25.70%.

Most endogenous AMPARs in the brain exist in complexes comprising GluA1/A2 or GluA2/A3 (Wenthold et al., 1996; Lu et al., 2009). To account for this, we transfected HEK293T cells with a combination of GluA1/A2 or GluA2/A3 at a ratio of 3:2. PORCN inhibited glutamate-induced currents in HEK293T cells expressing GluA1/A2 (Figure 1G) and GluA2/A3 (Figure 1H) by 67.88 ± 23.54% and 85.80 ± 19.71%, respectively. The coexpression of stargazin together with GluA1/A2 and GluA2/A3 was not associated with a PORCN-related inhibitory effect, as percentage inhibition was 64.55 ± 12.99% for GluA1/A2 and 42.87 ± 18.56% for GluA2/A3 (Figures 1I,J). Thus, PORCN inhibited glutamate-induced currents in a subunit- and stargazin-independent manner in transfected HEK293T cells.

### PORCN Suppressed the Surface Delivery of GluA1, GluA2, and GluA3 in Transfected HEK293T Cells and Cultured Hippocampal Neurons

**FIGURE 2:**
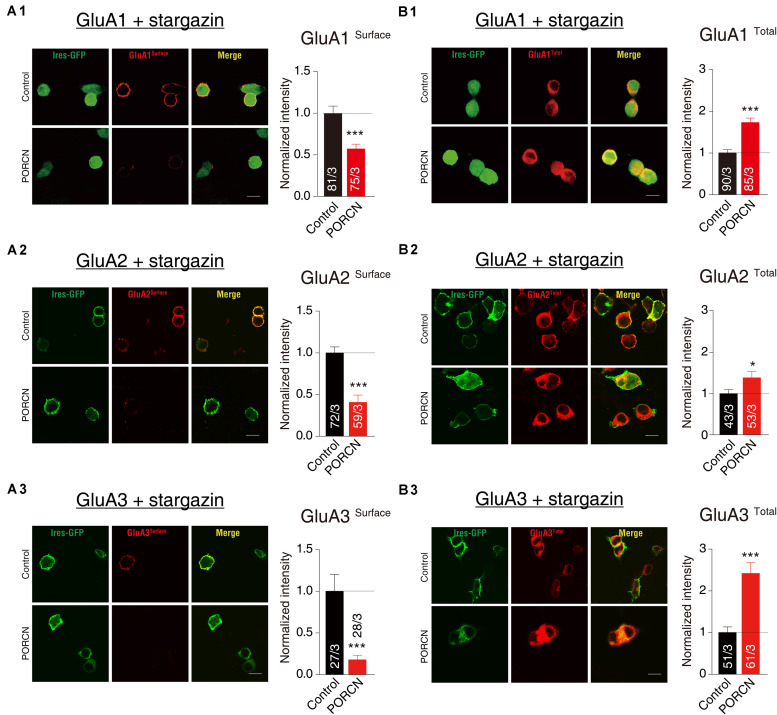
The overexpression of PORCN suppressed the surface expression of GluA1, GluA2, and GluA3 in transfected HEK293T cells. **(A)** Representative images (left) and quantification of the puncta intensity (right) of the surface expression of GluAs in HEK293T cells expressing GluA1 and stargazin **(A1)**, GluA2 and stargazin **(A2)**, or GluA3 and stargazin **(A3)** and transfected with either PORCN or a control plasmid. **(B)** Representative images (left) and quantification of the puncta intensity (right) of the total expression of GluAs in HEK293T cells expressing GluA1 and stargazin **(B1)**, GluA2 and stargazin **(B2)**, or GluA3 and stargazin **(B3)** and transfected with either PORCN or a control plasmid. The white lines in the images represent scale bars (scale bars = 10 μm). All summary graphs show means ± SEMs; statistical comparisons were performed with a student’s *t*-test (*p < 0.05; ***p < 0.001).

To investigate whether the PORCN-mediated inhibition of AMPAR-mediated currents was due to a reduction in surface AMPAR levels, we used quantitative immunocytochemistry to measure total and cell surface GluA protein levels in permeabilized and non-permeabilized transfected HEK293T cells, respectively. PORCN overexpression significantly reduced surface expression levels of GluA1, GluA2, and GluA3 in cells cotransfected with stargazin as indicated by decreased signals for the antibody against an extracellular epitope in non-permeabilized HEK293T cells. Signals decreased by 42.75 ± 11.09%, 59.30 ± 11.05%, and 82.11 ± 20.47%, respectively (Figures 2A1–A3). In contrast, immunostaining signals in permeabilized HEK293T cells for total GluA1, GluA2, and GluA3 levels were significantly higher in PORCN-transfected cells than in corresponding control cells (Figures 2B1–B3). This finding ruled out the possibility that the PORCN-mediated inhibition of surface GluA expression was due to a reduction in the expression of total AMPAR proteins. Thus, PORCN appeared to inhibit the plasma membrane delivery of AMPAR subunits (GluA1, GluA2, and GluA3) while increasing the total expression of these subunits in transfected HEK293T cells.

**FIGURE 3:**
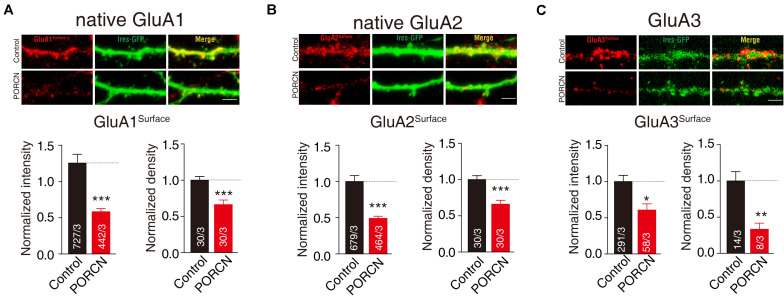
PORCN suppressed the surface expression of GluA subunits of AMPARs in cultured neurons. **(A)** Representative images and quantification of the intensity and density of the surface expression of native GluA1. **(B)** Representative images and quantification of the intensity and density of the surface expression of native GluA2. **(C)** Representative images and quantification of the intensity and density of the surface expression of GluA3 in neurons transfected with N-flag tagged GluA3. The white lines in the images represent scale bars (scale bars = 10 μm). All summary graphs show means ± SEMs; statistical comparisons were performed with a student’s t-test (*p < 0.05; **p < 0.01; ***p < 0.001).

To further determine whether PORCN suppressed the surface expression of GluA subunits of AMPARs in cultured neurons, we transfected hippocampal neurons with plasmid encoding PORCN together with plasmid encoding GFP plasmids. Then, we labeled surface GluA1 and GluA2 subunits using the antibody against extracellular epitopes in transfected neurons and measured surface GluA1 and GluA2 levels using quantitative immunocytochemistry. The intensity of surface GluA1 and GluA2 puncta in non-permeabilized neurons decreased by 67.32 ± 15.92% and 51.12 ± 10.15%, respectively, while their density decreased by 33.94 ± 8.224% and 34.26 ± 7.720%, respectively (Figures 3A,B). Thus, the overexpression of PORCN significantly reduced surface GluA1 and GluA2 levels. As there were no antibodies suitable for labeling surface GluA3 levels in neurons, so we transfected the hippocampus neurons with GluA3 with a flag tag in the N terminal and labeled the surface signal using the antibody against the flag tag. The intensity of GluA3-transfected hippocampus neurons decreased by 39.44 ± 19.70%, while their density decreased by 66.95 ± 18.33% (Figure 3C). Thus, as was the case with GluA1 and GluA2, the overexpression of PORCN significantly decreased surface GluA3 levels. Overall, our data suggested that PORCN suppressed the surface expression of GluA1, GluA2, and GluA3 subunits of AMPARs in cultured neurons.

### The ATD and CTD of AMPAR Subunits Are Not Required for the PORCN-Mediated Inhibition of AMPAR Delivery to the Plasma Membrane

Next, we investigated which AMPAR regions were required for the PORCN-mediated inhibition of AMPAR plasma membrane delivery. The removal of the LBD or transmembrane domain of AMPARs results in a complete loss of receptor function, so we focused the analysis on the ATD and CTD regions of AMPARs. To this end, we constructed thirteen plasmids expressing full or mutated versions of GluA1, GluA2, or GluA3. These plasmids had the ATD and various CTDs or the amino acid 824 from the CTD deleted. To delete the amino acid 824 from the CTD, we removed two serine phosphorylation sites and the PDZ-binding domain (Granger et al., 2013). In addition, we constructed a mutated version of GluA3 with KSRAESKRMKLTK (MPR) deleted, as this is known to disrupt the palmitoylation site and abolish the interaction with N4.1 in GluA1 (Lin et al., 2009; Supplementary Figure S1A). We generated GluA2-ΔATD and GluA3-ΔATD deletion constructs according to our previously reported protocol for GluA1-ΔATD construction (Wei et al., 2016, 2017). We generated CTD deletion constructs using strategies similar to those adopted previously for GluA1 (Sheng et al., 2018). We fused all GluA-ΔATD constructs with a flag tag immediately downstream from the signal peptide separated by a GQG spacer, and we fused GluA CTD deletion constructs with an HA tag at the extreme C-terminus separated by a GQG spacer. Then, we measured the ligand-gated currents of cells expressing these GluA mutants. As expected, glutamate elicited detectable inward currents in all cells coexpressing GluA and stargazin, except for HEK293T cells expressing GluA3-ΔC with four other amino acids, leaving only “EFCY” remaining in the AMPAR’s cytoplasmic tail (Supplementary Figure S1B). To analyze the contribution of the GluA3 CTD, we used the GluA3-ΔMPR deletion construct to complement the GluA3-Δ824 mutant in the following function assay.

**FIGURE 4:**
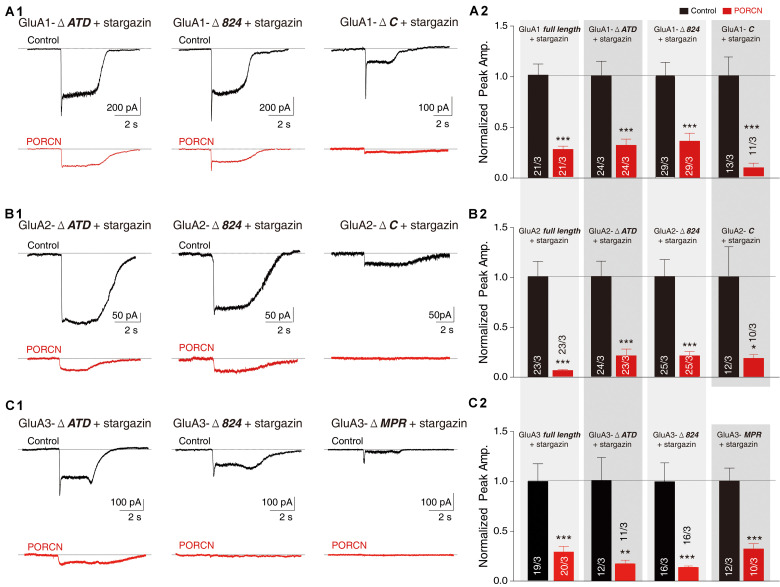
The ATD and CTD of GluAs are not required for the inhibitory effect of PORCN on glutamate-induced currents. **(A)** Representative traces **(A1)** and summary graphs **(A2)** of the normalized peak amplitudes of 10 mM glutamate-induced currents in HEK293T cells transfected with full-length GluA1 or GluA1 deletion constructs (GluA1-ΔATD, GluA1-Δ824, and GluA1-ΔC), stargazin, and either PORCN or a control plasmid. **(B)** Representative traces **(B1)** and summary graphs **(B2)** of the normalized peak amplitudes of 10 mM glutamate-induced currents in HEK293T cells transfected with full-length GluA2 or GluA2 deletion constructs (GluA2-ΔATD, GluA2-Δ824, or GluA2-ΔC), stargazin, and either PORCN or a control plasmid. **(C)** Representative traces **(C1)** and summary graphs **(C2)** of the normalized peak amplitudes of 10 mM glutamate-induced currents in HEK293T cells transfected with full-length GluA3 or GluA3 deletion constructs (GluA3-ΔATD, GluA3-Δ824, and GluA3-ΔMPR), stargazin, and either PORCN or a control plasmid. All summary graphs show means ± SEMs; statistical comparisons were performed with a student’s *t*-test (*p < 0.05; **p < 0.01; ***p < 0.001).

**FIGURE 5:**
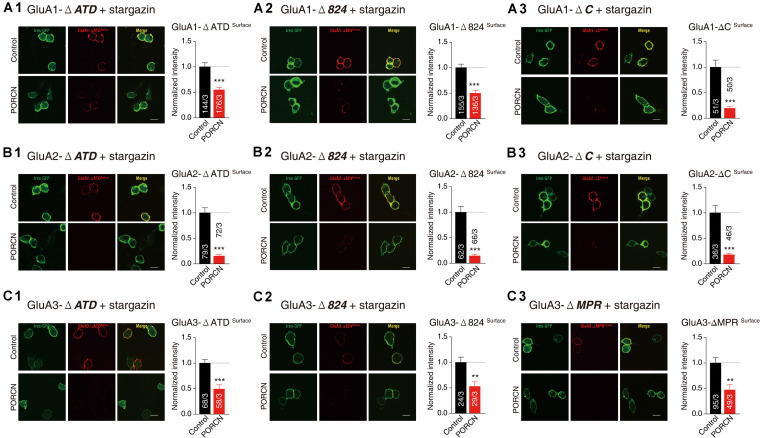
The ATD and CTD of GluAs are not required for the inhibitory effect of PORCN on the membrane expression of AMPARs in transfected HEK cells. **(A)** Representative images and quantification of the puncta intensity of the surface expression of GluA1 deletion constructs in HEK293T cells expressing GluA1 deletion constructs (**A1**: GluA1-ΔATD, **A2**: GluA1-Δ824, **A3**: GluA1-ΔC) and stargazin and transfected with either PORCN or a control plasmid. **(B)** Representative images and quantification of the puncta intensity of the surface expression of GluA2 deletion constructs in HEK293T cells expressing GluA2 deletion constructs (**B1**: GluA2-ΔATD, **B2**: GluA2-Δ824, **B3**: GluA2-ΔC) and stargazin and transfected with either PORCN or a control plasmid. **(C)** Representative images and quantification of the puncta intensity of the surface expression of GluA3 deletion constructs in HEK293T cells expressing GluA3 deletion constructs (**C1**: GluA3-ΔATD, **C2**: GluA3-Δ824, **C3**: GluA3-ΔMPR) and stargazin and transfected with either PORCN or a control plasmid. All summary graphs show means ± SEMs; statistical comparisons were performed with a student’s *t*-test (**p < 0.01; ***p < 0.001).

Our results demonstrated that glutamate-induced currents were significantly higher in control cells expressing stargazin and GluA1/2/3-ΔATD constructs and in control cells expressing the GluA1/2/3 CTD deletion constructs GluA1/2-ΔC, GluA1/2/3-Δ824, and GluA3-ΔMPR than in corresponding cells coexpressing PORCN. Percentage inhibitions for GluA1-ΔATD (69.87 ± 15.46%), GluA1-Δ824 (65.66 ± 15.35%), GluA1-ΔC (89.81 ± 19.65%), GluA2-ΔATD (79.53 ± 17.53%), GluA2-Δ824 (83.63 ± 19.53%), GluA2-ΔC (81.52 ± 31.68%), GluA3-ΔATD (82.47 ± 25.50%), GluA3-Δ824 (86.10 ± 18.90%), and GluA3-ΔMPR (67.98 ± 15.30%) have been shown in Figures 4A–C. Consistent with these results, the surface expression of AMPARs in non-permeabilized HEK293T cells transfected with GluA1/2/3-ΔATD or CTD deletion constructs was significantly suppressed by the overexpression of PORCN. Percentage inhibitions for GluA1-ΔATD (45.22 ± 8.90%), GluA1-Δ824 (50.15 ± 8.97%), GluA1-ΔC (80.55 ± 14.46%), GluA2-ΔATD (84.74 ± 10.79%), GluA2-Δ824 (85.62 ± 11.50%), GluA2-ΔC (82.23 ± 12.84%), GluA3-ΔATD (50.73 ± 10.06%), GluA3-Δ824 (52.84 ± 16.18%), and GluA3-ΔMPR (47.09 ± 13.62%) have been given in Figures 5A–C.

**FIGURE 6:**
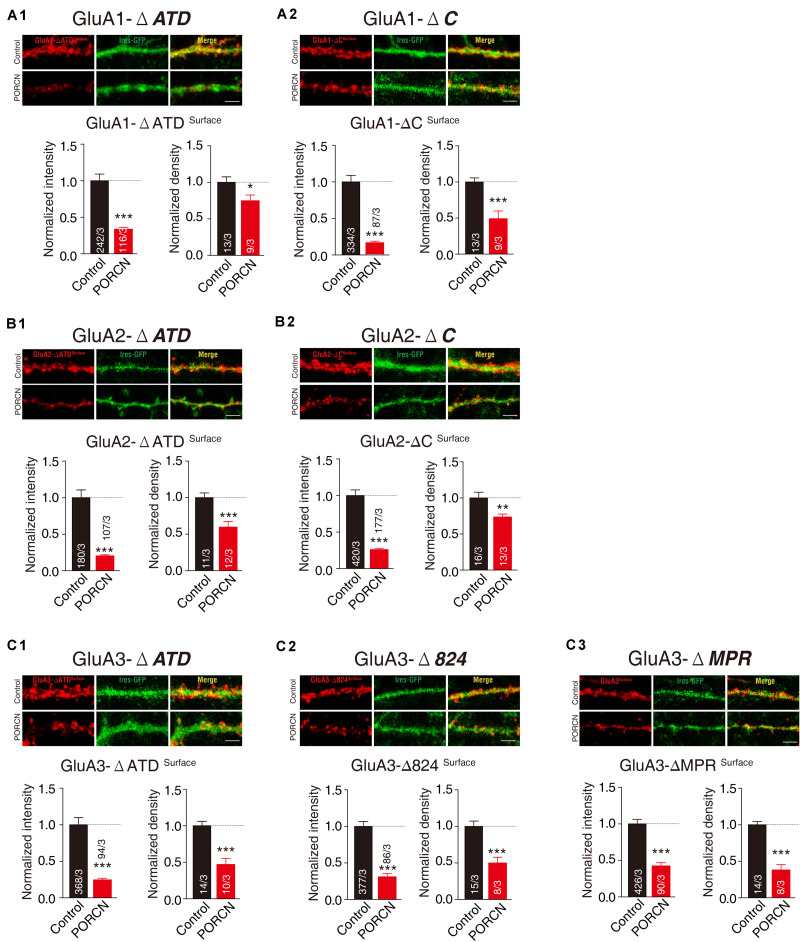
The ATD and CTD of GluAs are not required for the inhibitory effect of PORCN on the surface expression of AMPARs in cultured neurons. **(A1,A2)** Representative images and quantification of the intensity and density of the surface expression of GluA1 deletion constructs (**A1**: GluA1-ΔATD, **A2**: GluA1-ΔC) in cultured neurons expressing GluA1 deletion constructs. **(B1,B2)** Representative images and quantification of the intensity and density of the surface expression of GluA2 deletion constructs (**B1**: GluA2-ΔATD, **B2**: GluA2-ΔC) in cultured neurons expressing GluA2 deletion constructs. **(C1–C3)** Representative images and quantification of the intensity and density of the surface expression of GluA3 full length and deletion constructs (**C1**: GluA3-ΔATD, **C1**: GluA1-Δ824, **C3**: GluA3-ΔMPR) in cultured neurons expressing GluA3 deletion constructs. The white lines in the images represent scale bars (scale bars = 5 μm). All summary graphs show means ± SEMs; statistical comparisons were performed with a student’s *t*-test (*p < 0.05; **p < 0.01; ***p < 0.001).

To determine whether PORCN suppressed the surface expression of the various fragments of AMPARs employed in cultured neurons, we transfected neurons with plasmid encoding PORCN, together with GluA1-ΔATD, GluA1-ΔC, GluA2-ΔATD, GluA2-ΔC, GluA3-ΔATD, GluA3-Δ824, or GluA3-ΔMPR and labeled the surface signal using the antibody against the flag tag in the N terminal of deletion constructs. Quantitative immunocytochemistry showed that the overexpression of PORCN suppressed the surface expression of all deletion constructs. Decreases in the intensity of GluA1-ΔATD (66.47 ± 13.30%), GluA1-ΔC (83.47 ± 17.66%), GluA2-ΔATD (79.84 ± 13.89%), GluA2-ΔC (74.20 ± 11.99%), GluA3-ΔATD (75.61 ± 19.31%), GluA3-Δ824 (69.23 ± 14.05%), GluA3-ΔMPR (57.72 ± 13.44%) varied. Decreases in the density of GluA1-ΔATD (25.37 ± 11.17%), GluA1-ΔC (51.10 ± 11.19%), GluA2-ΔATD (40.54 ± 10.29%), GluA2-ΔC (26.60 ± 9.540%), GluA3-ΔATD (53.14 ± 10.26%), GluA3-Δ824 (50.29 ± 11.66%), GluA3-ΔMPR (62.02 ± 8.041%) also varied (Figures 6A1,A2,B1,B2,C1–C3). Taken together, our results demonstrated that neither the ATD nor the CTD of AMPAR subunits GluA1, GluA2, and GluA3 were required for the PORCN-mediated functional inhibition of AMPARs.

### The Interaction of PORCN With AMPARs Was Independent of Their ATD or CTD

**FIGURE 7:**
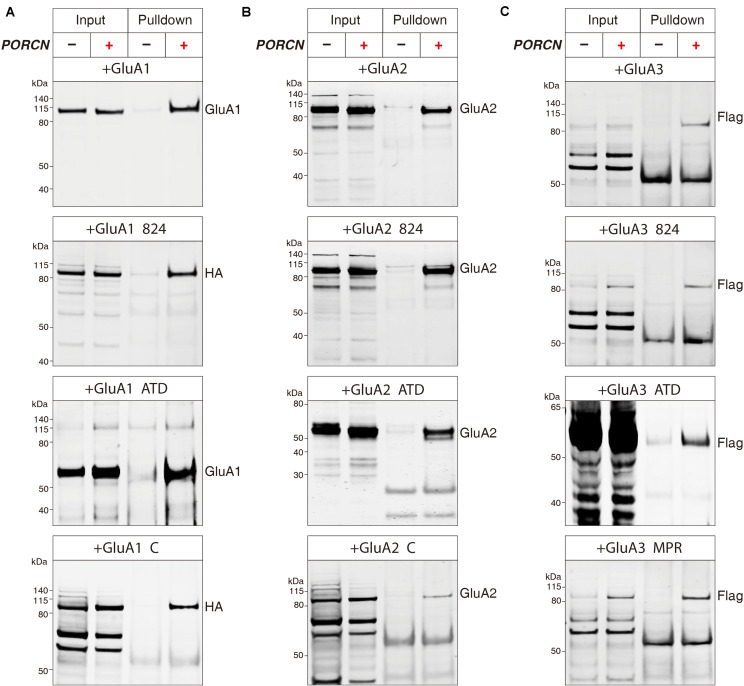
The binding of PORCN to AMPARs is independent of the AMPAR ATD or CTD. **(A)** Pulldown of GluA1, GluA1-ΔATD, GluA1-Δ824, and GluA1-ΔC expressed in transfected HEK293T cells together with the pulldown of myc-tagged PORCN with an anti-myc antibody. **(B)** Pulldown of GluA2, GluA2-ΔATD, GluA2-Δ824, and GluA2-ΔC expressed in transfected HEK293T cells together with the pulldown of myc-tagged PORCN with an anti-myc antibody. **(C)** Pulldown of GluA3, GluA3-ΔATD, GluA3-Δ824, and GluA3-ΔMPR expressed in transfected HEK293T cells together with the pulldown of myc-tagged PORCN with an anti-myc antibody.

The PORCN-mediated functional inhibition of AMPARs is thought to be caused by an interaction of PORCN with these receptors (Schwenk et al., 2012; Erlenhardt et al., 2016). We used immunoprecipitation assays to search for regions in AMPAR subunits that interacted with PORCN. We used an anti-myc antibody to immunoprecipitate myc-PORCN in HEK293T cells transfected with PORCN and wild-type or deletion mutants of GluA1-3 subunits. First, we determined whether PORCN coimmunoprecipitated with full-length and mutant GluA1. To this end, we used anti-GluA1 to detect the wild-type and GluA1-ΔATD mutant and an anti-HA antibody to detect GluA1-CTD deletion proteins, because the anti-GluA1 antibody used in this study recognized CTD regions that were absent from GluA1-CTD deletion constructs. Findings showed that Myc-tagged PORCN coimmunoprecipitated with full-length GluA1 when both PORCN and GluA1 were expressed (Figure 7A). Furthermore, PORCN coimmunoprecipitated with GluA1-ΔATD and GluA1 CTD mutants GluA1-Δ824 and GluA1-ΔC in transfected HEK293T cells (Figure 7A).

We performed similar experiments with GluA2 using an anti-GluA2 antibody that recognized the transmembrane region of GluA2 present in all GluA2 deletion constructs. GluA2-Δ824, GluA2-ΔATD, GluA2-ΔC, and the full-length GluA2 protein bound to myc-PORCN (Figure 7B). The results of similar experiments performed with GluA3 using an anti-flag antibody revealed that GluA3-Δ824, GluA3-ΔATD, and GluA3-ΔMPR as well as the full-length GluA3 protein bound to myc-PORCN (Figure 7C). Collectively, our data indicated that neither the ATD nor the CTD were involved in the interaction of PORCN with AMPARs, which suggested that other regions in AMPARs, such as the LBD and transmembrane domains, might be involved.

## Discussion

The trafficking of AMPARs to the plasma membrane determines the synaptic strength at excitatory synapses, and auxiliary subunits are key regulators of the intracellular and membrane delivery of AMPARs. PORCN, an auxiliary subunit of AMPARs, controls surface AMPAR levels in both transfected heterologous cells and in neurons (Schwenk et al., 2012; Erlenhardt et al., 2016). Here, we demonstrated that PORCN inhibits the ligand-gated currents and surface expression levels of GluA1, GluA2, and GluA3 in transfected HEK293T cells. This finding of subunit independence supported the previous finding that the inactivation of PORCN in hippocampal neurons reduced the total levels of GluA1, GluA2, and GluA3 (Erlenhardt et al., 2016). This inhibition required neither the ATD nor the CTD of these AMPAR subunits. Moreover, the interaction of PORCN with AMPARs was independent of the ATD and CTD of these AMPAR subunits. Thus, PORCN inhibited the function of AMPARs in a subunit-independent manner that did not involve the ATD or CTD of AMPARs.

Similar to the inhibitory effect of ABHD6, another auxiliary subunit of AMPARs (Schwenk et al., 2012; Wei et al., 2016, 2017), the inhibitory effect of PORCN on cell surface levels of GluA1, GluA2, and GluA3 in transfected HEK293T cells does not require the presence of either stargazin (γ-2, Figure 1C) or γ-8 (Erlenhardt et al., 2016). In contrast, the expression of CNIH-2, another auxiliary AMPAR subunit, in HEK cells slows the deactivation of AMPARs comprising GluA1, A2, or their combination; however, γ-8 expression reverses the effect of CNIH-2 on GluA2-containing AMPARs but not GluA1 homomers (Herring et al., 2013). Thus, multiple classes of auxiliary AMPAR proteins can mediate AMPAR trafficking to the plasma membrane.

Our data demonstrated that the ATD and CTD of AMPAR subunits GluA1, GluA2, and GluA3 were not required for the PORCN-mediated inhibition of AMPAR function or for the PORCN–AMPAR interaction. The ATD and CTD of AMPARs play substantial roles in the membrane trafficking of this receptor (Xia et al., 2002; Granger et al., 2013). Notably, multiple sites or regions in the CTD of AMPARs undergo protein modifications, such as nitrosylation, palmitoylation, ubiquitination, and phosphorylation/dephosphorylation [see (Diering and Huganir, 2018) for a detailed review]. A sophisticated molecular replacement strategy has been used to show that the PDZ binding motif in the AMPAR CTD is crucial for the synaptic delivery of AMPARs to the postsynaptic plasma membrane during both the basal state and long-term potentiation (Sheng et al., 2018). The interaction of other auxiliary subunits with the AMPAR CTD is essential. For example, ABHD6 reduces the surface expression levels of AMPARs in heterologous cells by binding to their cytoplasmic region (Wei et al., 2016, 2017). Another recent report indicated that the AMPAR’s ATD is involved in AMPAR trafficking. In the AMPAR complete knockout background, GluA1 or GluA2 expression resulted in the full or partial restoration of AMPAR-mediated synaptic transmission in Schaffer collateral pathways, while the expression of corresponding ATD deletion constructs did not rescue this transmission (Diaz-Alonso et al., 2017; Watson et al., 2017).

The exact molecules mediating this GluA-ATD interaction are unknown, but promising proteins associated with the GluA-ATD, including neuronal pentraxins, have been reported (O’Brien et al., 1999, 2002; Sia et al., 2007; Chang et al., 2010; Gu et al., 2013; Pelkey et al., 2015; Lee et al., 2017). In this study, AMPAR subunits, even in the absence of the ATD or CTD (for example, GluA-ΔC and GluA-ΔATD), constituted functional receptors in transfected HEK293T cells as indicated by their surface expression and capability to mediate glutamate-induced currents. Interestingly, PORCN inhibited the membrane expression of AMPARs and ligand-gated currents mediated by GluA CTD deletion constructs and GluA-ΔATD. This inhibition was not due to an effect on expression levels, because quantitative immunoblotting showed that the expression levels of these AMPAR mutants were not reduced to those of full-length AMPARs. This finding agreed with previous work, which showed that normal AMPAR-mediated synaptic transmission followed the replacement of endogenous AMPARs with various GluA1 CTD deletion mutants (Δ824, ΔMPR, or ΔC) in Cre-expressing Gria1-3fl/fl hippocampal CA1 neurons (Granger et al., 2013). Our data suggested that multiple mechanisms might act independently to regulate the processes through which AMPARs are delivered to the membrane.

Our patch clamp recording, immunostaining, and immunoprecipitation results indicated that the site at which AMPARs interact with PORCN was not located in the ATD or CTD and that it may be located in the LBD or transmembrane regions. Thus, the exact region in which PORCN and AMPAR subunits functionally interact remains to be identified. This issue must be addressed through further systematic molecular and cellular biology studies.

## Data Availability Statement

The original contributions presented in the study are included in the article/Supplementary Material, further inquiries can be directed to the corresponding authors.

## Author Contributions

MWe, LY, and CZ designed the research. MWe and LY performed the research. MWe, MWa, JW, FS, YW, MS, SW, MeL, HW, MiL, WL, and YG analyzed the data. CZ and LY wrote the manuscript. All authors contributed to the article and approved the submitted version.

## Conflict of Interest

The authors declare that the research was conducted in the absence of any commercial or financial relationships that could be construed as a potential conflict of interest.
