# Supplementary material for: PORCN Negatively Regulates AMPAR Function Independently of Subunit Composition and the Amino-Terminal and Carboxy-Terminal Domains of AMPARs
Source: Front Cell Dev Biol. 2020 Aug 25;8:829. doi: 10.3389/fcell.2020.00829 (PMC7477090; doi:10.3389/fcell.2020.00829)
Supplement: Supplementary file 1 [file Image_1.pdf]

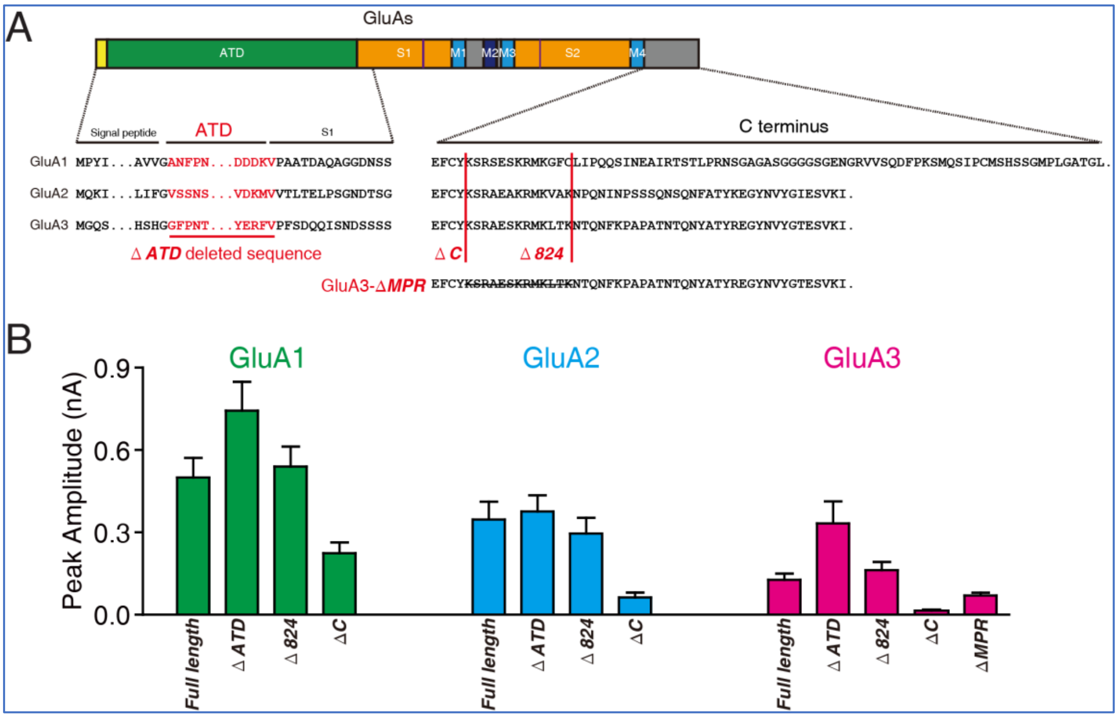

Supplemental Figure 1 Construction and verification of different GluA1-3 deletion constructs.

A. Schematic diagram of GluAs and the amino acid sequences of different GluA1-3 deletion constructs (GluA1-Δ824: ended with MKGFC, GluA1-ΔATD: delete the sequence between ANFPN and DDDKV, GluA1-ΔC: ended with EFCY, GluA2-Δ824: ended with MKVAK, GluA2-ΔATD: delete the sequence between VSSNS and VDKMV, GluA2-ΔC: ended with EFCY, GluA3-Δ824: ended with MKLTK, GluA3-ΔATD: delete the sequence between GFPNT and YERFV, GluA3-ΔC: ended with EFCY, and GluA3-ΔMPR： delete the sequence KSRAESKRMKLTK.

B. Summary graphs of the peak amplitudes of 10 mM glutamate-induced currents in HEK293T cells transfected with GluA1, GluA1-Δ824, GluA1-ΔATD, GluA1-ΔC, GluA2, GluA2-Δ824, GluA2-ΔATD, GluA2-ΔC, GluA3, GluA3-Δ824, GluA3-ΔATD, GluA3-ΔC, and GluA3-ΔMPR together with stargazin.
